# Male and Female Rats Have Different Physiological Response to High-Fat High-Sucrose Diet but Similar Myocardial Sensitivity to Ischemia-Reperfusion Injury

**DOI:** 10.3390/nu13092914

**Published:** 2021-08-24

**Authors:** Natacha Fourny, Carole Lan, Monique Bernard, Martine Desrois

**Affiliations:** Aix Marseille University, CNRS, CRMBM, 13005 Marseille, France; carole.lan@univ-amu.fr (C.L.); monique.bernard@univ-amu.fr (M.B.); martine.desrois@univ-amu.fr (M.D.)

**Keywords:** sex differences, prediabetes, myocardial ischemia-reperfusion injury, high-fat high sucrose diet, energy metabolism

## Abstract

Prediabetes is a strong predictor of type 2 diabetes and its associated cardiovascular complications, but few studies explore sexual dimorphism in this context. Here, we aim to determine whether sex influences physiological response to high-fat high-sucrose diet (HFS) and myocardial tolerance to ischemia-reperfusion injury. Male and female Wistar rats were subjected to standard (CTRL) or HFS diet for 5 months. Then, ex-vivo experiments on isolated perfused heart model were performed to evaluate tolerance to ischemia-reperfusion injury. HFS diet induced fasting hyperglycemia and increased body fat percent to a similar level in both sexes. However, glucose intolerance was more pronounced in female HFS. Cholesterol was increased only in female while male displayed higher level of plasmatic leptin. We observed increased heart weight to tibia length ratio only in males, but we showed a similar decrease in tolerance to ischemia-reperfusion injury in female and male HFS compared with respective controls, characterized by impaired cardiac function, energy metabolism and coronary flow during reperfusion. In conclusion, as soon as glucose intolerance and hyperglycemia develop, we observe higher sensitivity of hearts to ischemia-reperfusion injury without difference between males and females.

## 1. Introduction

According to the American Diabetes Association, prediabetes can be defined as elevated fasting plasma glucose 100–125 mg/dL (5.6–6.9 mmol/L), 2-h plasma glucose 140–199 mg/dL (7.8–11.0 mmol/L), or HbA1c 5.7–6.4% (39–46 mmol/mol) [1,2]. This is a reversible condition preceding well-established type 2 diabetes [3], highly related to sedentary lifestyle and consumption of high fat and/or high sucrose diet. The pre-diabetic condition already affects more than 33% of the American population and is becoming a real public healthcare issue, requiring more investigation and prevention [4]. Indeed, it is estimated that 5 to 10% of the prediabetic population will develop type 2 diabetes annually, while the risk in the normoglycemic population is around 0.7% [1].

Prediabetic patients also are at high risk of developing vascular and metabolic alterations promoting the occurrence of cardiovascular (CV) events [5,6]. People without type 2 diabetes but with the highest post-challenge blood glucose level have 27% greater risk for CV diseases than patients with the lowest post-challenge blood glucose level [7]. Recently, more attention has been paid to personalized medicine and particularly to the effect of sex [8]. Indeed, type 2 diabetes affects differently men and women regarding the associated CV complications. For example, the risk of myocardial infarction has been described to be higher in type 2 diabetic women than in type 2 diabetic men [9]. Importantly, differences between men and women have been reported before the onset of type 2 diabetes. Women have a greater deterioration of the CV system than men during the transition from normoglycemia to type 2 diabetes [10], and demonstrate higher endothelial, thrombotic, fibrinolytic factors [11,12]. However, few studies explore sex differences during the development of prediabetes and its effect on the myocardial tolerance to ischemia-reperfusion.

While more is known about the pathophysiological mechanisms involved in the CV complications associated with type 2 diabetes, little is understood in prediabetes. Nevertheless, some studies have revealed that energy production pathway could be involved in the development of micro and macro-vascular alterations in the prediabetic population [13,14]. Szucs et al., recently performed a proteomic analysis and showed a significant change in mitochondria-, apoptosis-, and oxidative stress-related proteins, in male rats fed with high-fructose diet [15]. A role of mitochondrial DNA methylation also has been highlighted in early-stage prediabetes but not in late-stage diabetes [16]. Hyperglycemia in prediabetes can lead to oxidative stress [17] and increase the sensitivity to CV events such as ischemia-reperfusion injury. We previously showed that five months high-fat high-sucrose (HFS) diet induced prediabetes in female Wistar rats [18], defined by mild hyperglycemia and glucose intolerance. Importantly, HFS increased oxidative stress after ischemia-reperfusion injury, characterized by eNOS uncoupling and higher malondialdehyde level. Mahat et al., also demonstrated that markers of oxidative stress such as malondialdehyde (MDA) were found to be significantly increased in prediabetic subjects as compared to control subjects [19]. However, no study has compared energy metabolism in parallel to cardiac function in prediabetic male and female rats before and after ischemia-reperfusion injury. Therefore, our objectives were to determine whether the sex influences the physiological response to HFS diet and to compare myocardial tolerance to ischemia-reperfusion injury in male and female Wistar rats. We hypothesized that impairment of energy metabolism plays a role in cardiac sensitivity to ischemia-reperfusion injury in prediabetes in association with oxidative stress. Thus, we simultaneously evaluated cardiac function, coronary flow and high energy compounds using phosphorus-31 magnetic resonance spectroscopy (^31^P-MRS). We also assessed markers of oxidative stress such as MDA and 8-isoPGF2α.

## 2. Materials and Methods

### 2.1. Animals

Seven-week-old female (*n* = 20) and male (*n* = 24) Wistar rats (Charles River, France) were housed in a ventilated rack cabinet with controlled temperature (22–24 °C), light-dark cycle of 12:12 h and multiple environmental enrichments. All procedures were approved by the animal experiment ethic committee of the University (APAFIS #10547-2017071009112930) and were carried out in accordance with the Directive 2010/63/EU of the European Parliament on the protection of animals used for scientific purposes.

### 2.2. Diet

Animals had access to food and water *ad libitum*. Female and male Wistar rats were both randomly divided into two groups of respectively ten and twelve animals. Control groups (female: F-CTRL, male: M-CTRL) were fed with standard diet (SAFE, A04C-10; Table 1); and HFS groups (female: F-HFS, male: M-HFS) were fed with high-fat high-sucrose diet (SAFE, U8978 v19; Table 1) for 5 months. Animal weight and food intake were recorded throughout the months.

### 2.3. Tolerance to Glucose, Arterial Blood Pressure and Amount of Adipose Tissues

After 5 months of diet, an intraperitoneal glucose tolerance test (IPGTT) was performed to evaluate glucose homeostasis. Animals were fasted for six hours, with free access to water during the entire experiment. Rats were weighed and the extremity of the tail was incised in order to obtain a small drop of blood, which was placed on the test strip of the blood nano-glucometer ACCU-CHECK Performa (Roche, Bale, Switzerland). The value obtained corresponds to the fasting blood glucose level (T0). Then a bolus of glucose was injected in the intraperitoneal cavity at the dose of 1 g/kg. Glycemia was measured 15, 30, 60, 90, 120 min after the injection of glucose.

Systolic and diastolic blood pressure were recorded non-invasively by the tail-cuff method (Bioseb, Chaville, France). The animals were lightly sedated with isoflurane (1%), the body temperature was maintained at 38.5 °C using a heating blanket, and blood pressure and heart rate were recorded (10 measurements). Averaged values for each rat were used for the subsequent statistical analysis.

At the time of sacrifice, amounts of subcutaneous and visceral adipose tissues were dissected and weighed. The sum of gonadal, mesenteric, retroperitoneal and perirenal fat was considered as visceral fat. Fat percent was determined using the following formula: ((subcutaneous + visceral adipose tissues) × 100)/total body weight.

### 2.4. Myocardial Tolerance to Ischemia-Reperfusion Injury

After 5 months of diet, rats were anesthetized by intraperitoneal injection of 90 mg/kg pentobarbital sodium. Hearts were weighed before cannulation and perfused in the Langendorff mode at a constant pressure of 100 mmHg as previously described [18].

**Figure 1:**
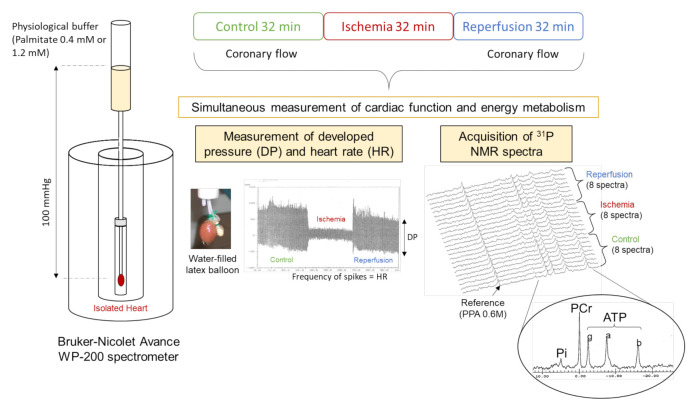
Schematic representation of the experimental protocol of ischemia-reperfusion on isolated perfused heart. Cardiac function was evaluated using a water-filled balloon related to a pressure transducer and inserted in the left ventricle. Energy metabolism was evaluated simultaneously using ^31^Phosphorus magnetic resonance spectroscopy.

The experimental protocol is summarized and represented in Figure 1. After stabilization, hearts were perfused for 24 min with a physiological Krebs-Henseleit buffer containing 0.4 mM palmitate, 3% albumin, 11 mM glucose, 3 U/L insulin, 0.8 mM lactate, and 0.2 mM pyruvate. Four minutes before low-flow ischemia, hearts were perfused with a physiological Krebs-Henseleit buffer containing 1.2 mM palmitate. Then hearts underwent a low-flow ischemia (0.5 mL/min/g wet wt) during 32 min with the same buffer. Finally, flow was restored entirely for 32 min with the physiological recirculating Krebs-Henseleit buffer containing 0.4 mM palmitate. The perfusates were continually gassed with a mixture of 95% O_2_ and 5% CO_2_ to maintain pH at 7.4. The buffer temperature was maintained at 37 °C during all the protocol. At the end of the experiments, hearts were freeze-clamped in liquid nitrogen and kept at −80 °C for further analysis.

### 2.5. Myocardial Function and Coronary Flow

A water-filled latex balloon was inserted into the left ventricle to record left ventricular developed pressure (DP) and heart rate (HR), as previously described [18]. The product of DP and HR was used as an index of cardiac function. During reperfusion, we calculated the percentage recovery between the pre-ischemic and post-ischemic cardiac function. Coronary flow was measured by collection of coronary effluent before and after ischemia (at 20 min and 80 min) and expressed in mL/min/g wet weight.

### 2.6. Myocardial Energy Metabolism

Evaluation of high-energy compounds is a good indicator of mitochondrial function and oxidative stress in the heart. To do so, perfused rat hearts were placed in a 20-mm magnetic resonance sample tube and inserted in a 4.7 Tesla magnet (Oxford instruments, Oxford, UK) interfaced with a Bruker-Nicolet Avance WP-200 spectrometer (Bruker, Karlsruhe, Germany). A series of eight ^31^P NMR spectra were recorded during each period of the experimental protocol to quantify phosphorus metabolites (Adenosine triphosphate: ATP, phosphocreatine: PCr and inorganic phosphate: Pi) and intracellular pH (pHi). Spectra were obtained by signal averaging 328 accumulations acquired during 4 min. Prior to Fourier transformation, the free induction decay was multiplied by an exponential function which generated a 20 Hz line broadening. The positions and areas of the resonances were determined using the AMARES software package (jMRUI; http://www.jmrui.eu/; accessed on 26 February 2018). Values for pHi were derived from the chemical shift of the Pi resonance taking into account the variations of temperature. Phenylphosphonic acid (0.6 M) in a glass capillary placed along the heart was used as an external reference for quantification and peaks areas were corrected for saturation. Cytosolic concentrations (mml/L) were calculated considering the intracellular volume and the tissue weight. [18,20,21].

### 2.7. Biochemical Analyses

In plasma, assay kits were used to determine glycemia (Randox Laboratories, Crumlin, Antrim, UK), free fatty acids (NEFA kit, Roche, Mannheim, Germany), leptin (Rat leptin ELISA kit, Crystal Chem, Netherlands), LDL and HDL cholesterol (Abcam, ab65390) and 8-iso-PGF2α (Direct 8-iso-PGF2α ELISA kit, Enzo Life Science, Lausen, Switzerland).

In hearts, malondialdehyde (MDA) and mitochondrial citrate synthase (CS) activity were evaluated (MAK085-1KT, Sigma-Aldrich; CS0720, Sigma-Aldrich; Saint-Louis, MO, USA) after ischemia-reperfusion injury.

### 2.8. Statistical Analyses

Data are graphically provided as means ± SEM of absolute values. GraphPad Prism software 5.0 (La Jolla, San Diego, CA, USA) was used for all statistical processing. Significant differences between groups were determined using two-way analysis of variance (ANOVA) with repeated measures over time for the time-dependent variables, followed by Bonferroni post-hoc test. One-way ANOVA was performed for the other parameters. A *p*-value of less than or equal to 0.05 was considered to indicate significant difference.

## 3. Results

### 3.1. Physiological Parameters

**Figure 2:**
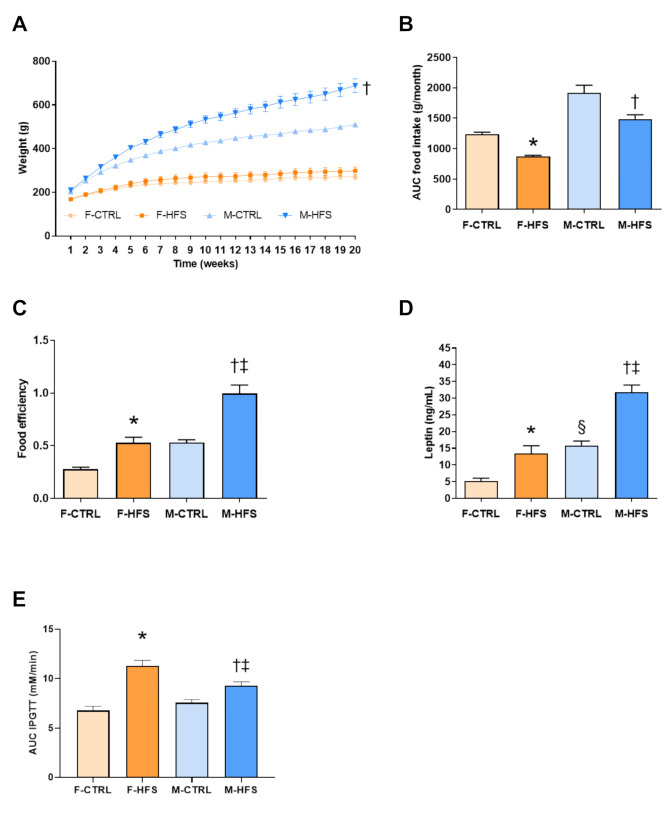
Weight of animals over time (**A**), food intake (**B**), food efficiency (**C**), leptin level (**D**), and area under the curve (AUC) of glycemia over time during the glucose tolerance test (**E**). Data are means ± SEM. Two-way ANOVA with repeated measures over time for the time-dependent variables followed by Bonferroni post-hoc test. One-way ANOVA was performed for the other parameters. * *p* < 0.05 F-HFS vs. F-CTRL; † *p* < 0.05 M-HFS vs. M-CTRL; ‡ *p* < 0.05 M-HFS vs. F-HFS; § *p* < 0.05 M-CTRL vs. F-CTRL. F-CTRL: female control; F-HFS: female high-fat high-sucrose; M-CTRL: male control; M-HFS: male high-fat high-sucrose.

From the 5th week, M-HFS exhibited higher weight than M-CTRL, whereas no difference was found between F-HFS and F-CTRL during the whole diet (Figure 2A). We found a lower food intake (Figure 2B) in F-HFS and M-HFS (*p* < 0.05 and *p* < 0.01 vs. respective controls) as well as a higher food efficiency (representing the weight gained over the food intake, Figure 2C) in F-HFS and M-HFS in comparison to respective controls (*p* < 0.05 and *p* < 0.001). Food efficiency was also higher in M-HFS in comparison with F-HFS (*p* < 0.001) indicating that males are more prone to gain weight than females. In line with these results, we showed an increase in plasma level of leptin at 5 months (Figure 2D) in F-HFS and M-HFS in comparison with respective controls (*p* < 0.001 and *p* < 0.05), as well as an increase in leptin level in M-HFS in comparison with F-HFS (*p* < 0.05). The IPGTT after 5 months of diet (Figure 2E) indicates glucose intolerance in M-HFS and F-HFS rats in comparison with their respective controls (*p* < 0.05 and *p* < 0.001). Interestingly, intolerance to glucose was more pronounced in F-HFS versus M-HFS (*p* < 0.05).

The other physiological parameters after 5 months of diet are shown in Table 2. Fasting blood glucose was significantly increased in F-HFS and M-HFS (*p* < 0.001 and *p* < 0.05) in comparison with control groups, without sex difference. HDL-cholesterol was similar between groups. However, LDL-cholesterol was significantly increased only in F-HFS in comparison with F-CTRL and M-HFS (*p* < 0.001 and *p* < 0.01). Free fatty acids were similar between groups. Fat mass was higher in both HFS groups in comparison with respectivecontrols (*p* < 0.01 and *p* < 0.05). Nevertheless, both visceral and subcutaneous adipose tissues were increased only in M-HFS in comparison with M-CTRL (*p* < 0.001 and *p* < 0.01). Heart weight, as well as the heart weight to tibia length ratio, were also significantly increased only in M-HFS versus M-CTRL (*p* < 0.001). Systolic and diastolic blood pressure were not different between the four groups. Finally, 8-iso-PGF2α level, an indicator of basal oxidative stress, was similar between groups.

### 3.2. Tolerance to Ischemia-Reperfusion Injury

#### 3.2.1. Myocardial Function and Coronary Flow

**Figure 3:**
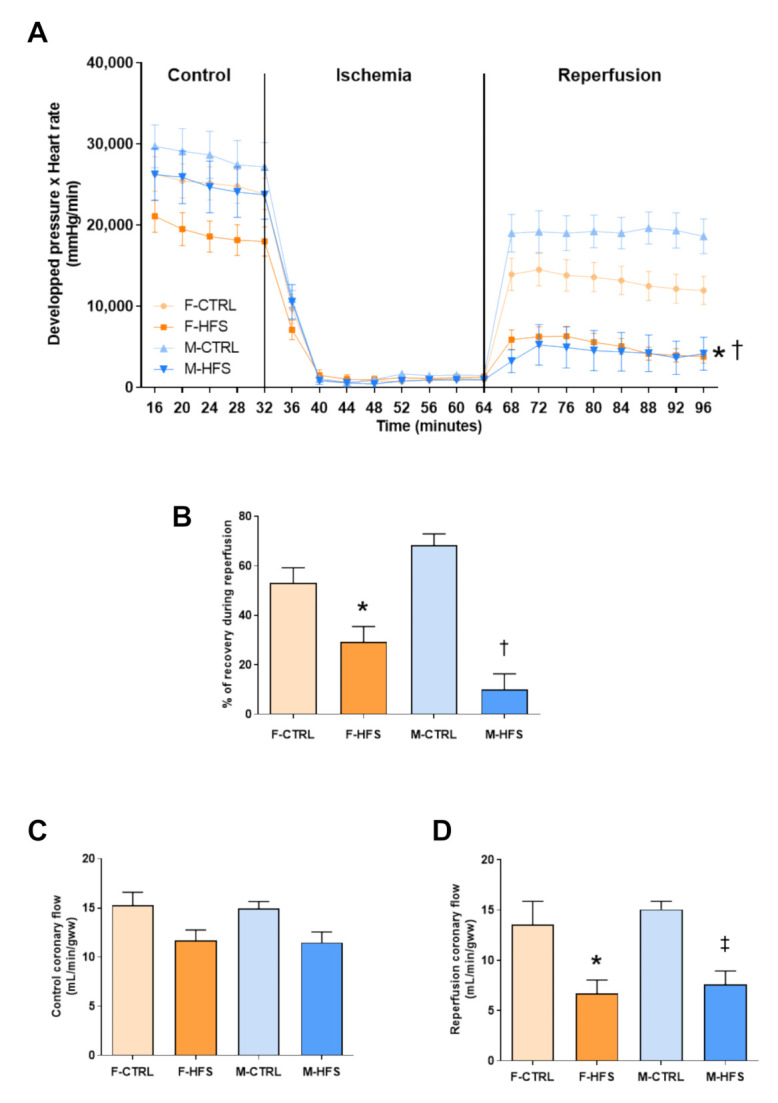
Myocardial function evaluated by the product of developed pressure and heart rate (**A**), percent of recovery during reperfusion (**B**), control coronary flow (**C**) and reperfusion coronary flow (**D**). Data are means ± SEM. Two-way ANOVA with repeated measures over time for the time-dependent variables followed by Bonferroni post-hoc test. One-way ANOVA was performed for the other parameters. * *p* < 0.05 F-HFS vs. F-CTRL; † *p* < 0.05 M-HFS vs. M-CTRL; ‡ *p* < 0.05 M-HFS vs. F-HFS.

Myocardial function (Figure 3A) was evaluated by the product of developed pressure and heart rate during the entire experimental protocol. Myocardial function was not different between groups during the control period, despite a tendency to a decrease in F-HFS. Myocardial function was significantly impaired in both F-HFS and M-HFS during reperfusion (*p* < 0.05 and *p* < 0.001 vs. respective controls). The percent of functional recovery during reperfusion (Figure 3B) was lower in F-HFS and M-HFS (*p* < 0.05 and *p* < 0.001 vs. respective controls), without sex difference.

Coronary flow was not significantly different between groups during the control period (Figure 3C), despite a trend toward decrease in both HFS groups. However, during reperfusion (Figure 3D), coronary flow was significantly decreased in F-HFS and M-HFS in comparison with controls (*p* < 0.05 and *p* < 0.01), without sex difference. This could indicate an impairment of endothelial function, participating in the decrease of cardiac function at reperfusion.

#### 3.2.2. Myocardial Energy Metabolism

**Figure 4:**
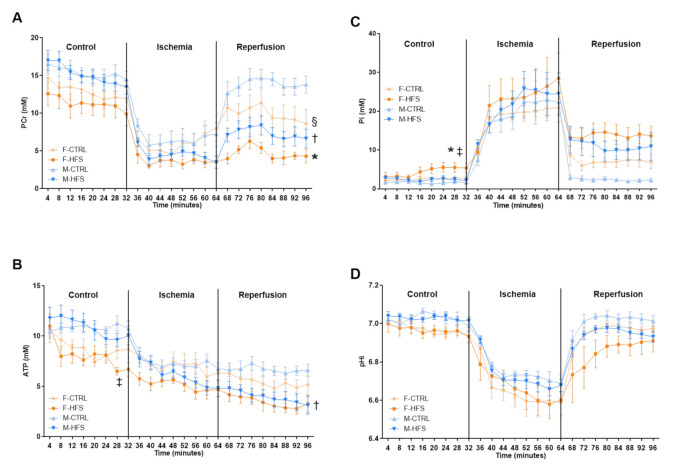
Energy metabolism. Kinetics of PCr (**A**), ATP (**B**), Pi (**C**) and pHi (**D**) during the experimental protocol. Data are means ± SEM. Two-way ANOVA with repeated measures over time for the time-dependent variables followed by Bonferroni post-hoc test. * *p* < 0.05 F-HFS vs. F-CTRL; † *p* < 0.05 M-HFS vs. M-CTRL; ‡ *p* < 0.05 M-HFS vs. F-HFS; § *p* < 0.05 M-CTRL vs. F-CTRL.

To understand the decrease in myocardial function observed in HFS groups, we evaluated kinetics of PCr, ATP, Pi and pHi, respectively represented in Figure 4. During the control period and ischemia, PCr (Figure 4A) was not different between groups. During reperfusion, PCr was significantly lower in F-HFS and M-HFS in comparison with the respective controls (*p* < 0.05 and *p* < 0.001), without sex difference. This could indicate a lower efficacity of mitochondria to produce energy. During the control period, ATP (Figure 4B) was also significantly decreased in F-HFS in comparison to M-HFS (*p* < 0.05) and during reperfusion, ATP was significantly decreased only in M-HFS versus M-CTRL (*p* < 0.01). Pi (Figure 4C) was significantly increased only in F-HFS during the control period in comparison with F-CTRL (*p* < 0.05) and M-HFS (*p* < 0.01). Finally, pHi (Figure 4D) was similar in the four groups during the whole experimental protocol.

#### 3.2.3. Cardiac MDA Content and CS Activity

**Figure 5:**
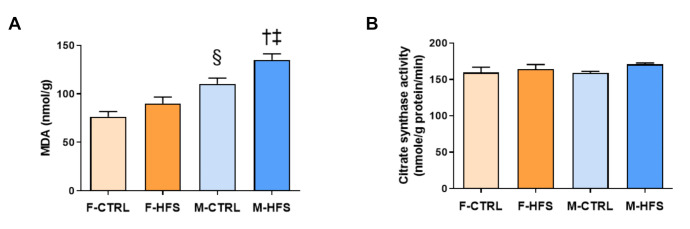
Cardiac malondialdehyde (**A**) and citrate synthase activity (**B**). Data are means ± SEM. One-way ANOVA was performed for both parameters. † *p* < 0.05 M-HFS vs. M-CTRL; ‡ *p* < 0.05 M-HFS vs. F-HFS; § *p* < 0.05 M-CTRL vs. F-CTRL.

The lipid peroxidation marker MDA (Figure 5A) was significantly higher in M-CTRL in comparison with F-CTRL (*p* < 0.01). Interestingly, HFS diet increased MDA after ischemia-reperfusion injury in M-HFS (*p* < 0.05) but not in F-HFS in comparison to respective controls. M-HFS also had higher MDA than F-HFS (*p* < 0.001). CS activity (Figure 5B) was assessed as one marker of the Krebs cycle activity and was similar in the four groups.

## 4. Discussion

According to the International Federation of Diabetes, prediabetes will affect more than 470 million people worldwide in 2030 [22]. Unhealthy diet and sedentary lifestyle highly contribute to the development of prediabetes, and importantly, prediabetes is a strong predictor of the development of type 2 diabetes and its associated cardiovascular complications [6]. Here, we aimed to determine whether the sex influences the physiological response to high-fat high-sucrose diet and the myocardial tolerance to ischemia-reperfusion injury.

### 4.1. Sex Influences the Physiological Response to High-Fat High-Sucrose Diet

Too much sugar or fat intake increase the risk of prediabetes, obesity and type 2 diabetes in both humans and rodents [23]. Interestingly, despite a long period of HFS diet, we found a significant increase in body weight only in male rats. The same observation has been made in other studies in which female did not gain weight and were more resistant to enriched diets [18,24,25]. Sex-specific regulation of the leptin pathway in response to the HFS diet may be responsible for the difference in weight gain between males and females. Here, leptin level was increased in both HFS groups but more markedly in M-HFS. In humans, Kratz M et al., showed that increased leptin level was related to a decrease in food intake [26]. In animal studies, male rats have a higher level of leptin in comparison to female rats under a high-fat diet [27,28], as found here. Leptin level is correlated with the amount of adipose tissue in the body [29]. Thus, in our study the increase in leptin could be related to higher amount of adipose tissue leading to lower food intake in M-HFS. Independently of the link between leptin and weight, it is important to mention the role of leptin on glucose metabolism, with a correlation between leptin and insulin levels. Several studies demonstrated the existence of an adipoinsular axis, characterized by leptin-inhibited insulin synthesis and secretion [30,31], promoting hyperglycemia, as shown in our model.

Sex differences have been reported in the development of prediabetes in terms of glucose homeostasis. Here we have shown that HFS diet induces the development of fasting hyperglycemia and glucose intolerance in HFS rats which is concordant with a previous study using the same diet in female rats [32]. However, we showed that HFS females had more pronounced glucose intolerance than HFS males with similar impaired fasting blood glucose. Human studies have also demonstrated higher association between lifestyle and glycemic outcomes in women than in men [33,34]. Women are also more prone to develop glucose intolerance, strongly associated to insulin resistance, while men are more prone to develop impaired fasting blood glucose [35]. The reasons for these differences could be due to hormones. Indeed, estrogen supplementation in postmenopausal women reduces fasting blood glucose levels [35]. In-vitro studies on human isolated pancreatic islet showed that estrogen improves pancreatic hormone secretion, increasing insulin secretion and decreasing glucagon secretion [36]. Saengsirisuwan et al., also showed that ovariectomized rats had lower glucose tolerance than non-ovariectomized females [37]. On the other hand, incretin hormone such as GLP-1 might be affected by sex. In prediabetes, women have a decreased relative response to GLP-1 (18 to 25%) compared with non-pre-diabetic women. In prediabetic men this relative response does not differ in comparison to healthy men [38]. Therefore, it would be interesting to consider this information in future studies.

As reported here, Petersson et al., also found no difference in plasmatic 8-isoPGF2α in subjects submitted to high-fat diets in the LIPGENE study [39]. We also found a higher level of LDL-Cholesterol in F-HFS in comparison with the other groups. In type 2 diabetic patients, LDL-Cholesterol management was shown to be worse in women than in men, as found in our work [40,41]. Some studies have explored the possible mechanistic interaction between estrogens and cholesterol. Liver estrogen signaling may contribute to sex-differences in atherosclerosis by promoting the hepatic steps of reverse cholesterol transport. Moreover, Peng et al., showed that sex-specific modification of the gut microbiota in response to high-fat diet could be associated with the different sensitivity of male and female mice to metabolic disorders [42]. Nevertheless, sex differences observed in prediabetes and diabetes are not yet fully understood and more studies should be conducted on this topic.

### 4.2. Sex Influences Cardiac Morphology and Function When Submitted to High-Fat High-Sucrose Diet

We report higher heart weight-to-tibia length ratio only in M-HFS in comparison to F-HFS, which could be an indicator of cardiac hypertrophy. This result is consistent with our previous work on female Wistar rats fed with HFS diet in which we did not observe an increase in heart/tibia ratio [32]. Cardiac hypertrophy has been explained in the literature by increased collagen levels or proliferation of cardiac fibroblasts [43,44,45]. Interestingly, Böhm C et al., showed sexual dimorphism in obesity-induced left ventricular hypertrophy in mice as we found. After 25 weeks of a high-fat diet, the authors showed a significantly greater increase in left ventricular mass in male mice than in female mice, associated with higher level of leptin, adiponectine and vaspin leading to proliferation of cardiac fibroblasts. At the same time, males had higher weight and fat mass than females [46]. Dworatzek et al., recently discussed this point and proposed a sex-mediated role of estrogen on collagen synthesis, which is inhibited in female cardiac fibroblasts but stimulated in males’ ones [47].

We also showed that cardiac function before ischemia was not impaired by HFS diet despite a strong declining trend in F-HFS. In agreement with others, we previously showed that HFS diet induces impaired cardiac function in female rats ex vivo characterized by lower developed pressure but without abnormalities in vivo [48,49,50]. Indeed, in vivo experiments showed no alteration of ejection fraction or fractional shortening that could indicate cardiac dysfunction. Importantly, we previously reported in our model higher myocardial perfusion, probably to compensate and maintain normal function. In more advanced models of type 2 diabetes like the GK rat, cardiac function and myocardial function (ejection fraction) are impaired both in vivo and ex vivo, showing the switch particularly occurring in females [18,51]. Impairment of energy metabolism could explain why F-HFS present a lower cardiac function in baseline conditions. Indeed, we found lower ATP and higher Pi contents in F-HFS group than in M-HFS, suggesting greater basal impairment of mitochondrial function. Manrique et al., observed increased β-Hydroxyacyl-coenzyme A activity and decreased CS activity in western diet-fed female mice, contributing to increased oxidative stress, reduced myocardial metabolic efficiency, and diastolic dysfunction [50]. Recent studies also highlighted the potential role of increased mitochondrial protein O-GlcNAcylation in mitochondrial dysfunction. O-GlcNAcylation is a post-translational modification which has been shown to have a role in cardiac hypertrophy, diabetes and cardiovascular diseases [52,53]. Ma et al., conducted O-GlcNAcomic and revealed more than 88 mitochondrial proteins targeted by O-GlcNAcylation [54]. However, to our knowledge no study explored the effect of sex on the O-GlcNAcylation process. Thus, future investigations should be conducted to determine if energy metabolism impairment in prediabetes is mediated by modification of protein O-GlcNAcylation.

### 4.3. Sex Does Not Influence the HFS Myocardial Tolerance to Ischemia-Reperfusion Injury

As mentioned before, prediabetic population is at high risk of cardiovascular complications, and particularly women who have an increased CV risk in comparison to men [55]. The DECODE study showed that impaired glucose tolerance of prediabetic individuals was associated with higher coronary disease and death [56]. However, few studies explore the effect of sex in this context and the molecular mechanisms involved are still poorly understood.

After ischemia-reperfusion injury, we found a strong impairment of myocardial function in both female and male rats fed with the HFS diet, in comparison with controls. Despite, sex differences in some physiological parameters, cardiac function and energy metabolism during the control period, cardiac function was similarly impaired in male and female HFS during reperfusion. The alteration of cardiac function at reperfusion can be explained here in part by impaired energy metabolism. The decrease in PCr and ATP in male and female HFS at reperfusion could be due to impaired mitochondrial respiratory chain or the activity of Krebs cycle enzymes. Recently, Chen et al., showed a decrease in the expression of complexes I, II and III of the mitochondrial respiratory chain, citrate synthase activity, total adenylated nucleotides, associated with impaired cardiac function in the hearts of high-fat fed male rats [57]. Here we found no difference between groups for the citrate synthase activity. This enzyme is the first enzyme of the Krebs cycle, but it is possible that other enzymes such as the isocitrate dehydrogenase are involved in the impairment of energy metabolism. A proteomic study on prediabetic animal (high-fat diet induced) identified a decrease in the expression of three proteins involved in energy metabolism (mitochondrial ATP synthase β subunit, adenylate kinase and creatine kinase) and the isocitrate dehydrogenase of the Krebs cycle [58]. An alteration of the mitochondrial respiratory chain is also possibly involved in the impairment of energy metabolism.

The alteration of cardiac function at reperfusion can also be partly explained by endothelial dysfunction, which has been described as early as in the pre-diabetes [14]. Here we found a strong alteration in coronary flow at reperfusion in both HFS groups. Alterations in vascular response to various pharmacological agents, hypertrophic vascular remodeling and a significant decrease in eNOS expression have been reported in male rats fed with a diet high in fat and/or sugar [45,50,59,60]. We have previously shown the involvement of eNOS uncoupling in the decrease of coronary flow during reperfusion in HFS females, in relation with a higher oxidative stress [32].

Here we showed that the lipid peroxidation marker MDA, was significantly higher in M-HFS in comparison with M-CTRL and F-HFS. MDA is one marker of lipid peroxidation and other markers would be interesting to measure in the future, like protein, DNA oxidation or antioxidant enzymes. Indeed, Liu et al., postulated that the system of regulation against cellular oxidative stress might differ between male and female rats, with lower production of antioxidant proteins in male under high-fat diet [61]. Sibouakaz et al., also reported increased plasmatic MDA, more importantly in male rabbit under high-fat diet than female [62]. However, these studies did not include the role of sex in oxidative stress after ischemia-reperfusion injury. In contrast, Fekete et al., showed that HSP72, a protective heat shock protein against cellular stress, was higher in female than in male Wistar rats before and after renal ischemic injury [63]. In a previous study we showed that female HFS rats had lower S-Glutathionylation of proteins, which is a reversible addition of glutathione to cysteine residues inactivating targeted proteins. In case of ischemia-reperfusion injury it can be a protective mechanism coping with irreversible oxidation. So, a decrease in S-Glutathionylation indicates increased oxidative stress [18].

## 5. Conclusions

In conclusion, five months of a high-fat high-sucrose diet induced prediabetes in males and female rats, with sex differences in weight gain, leptin level, glucose tolerance and heart/tibia ratio. HFS diet also decreased myocardial tolerance to ischemia-reperfusion in both sexes, characterized by impaired energy metabolism, cardiac function and coronary flow during reperfusion without sex differences. This work confirms the deleterious impact of early-stage prediabetes and the role of mitochondrial dysfunction in myocardial ischemia-reperfusion-induced injury. For the sake of perspective, it would be interesting to perform a study with ovariectomized rats and address this question: would females be more sensitive to ischemia-reperfusion injury than males without estrogen?

## Figures and Tables

**Table 1:** Nutritional composition of control (CTRL) and high-fat high sucrose (HFS) diets.

%	CTRL Diet	HFS Diet
Nitrogen free extract	60.4	37.23
Of which starch	43.5	14.52
Of which Sugars	3.2	20.25
Crude proteins	16.1	19.84
Crude fat	3.1	35.92
Crude ash	4.6	4.2
Crude fiber	3.9	<0.5
Moisture	11.9	2.8
Minerals/Vitamins	3.9	7.5

**Table 2:** Physiological parameters of animals after 5 months of CTRL or HFS diet.

	F-CTRL	F-HFS	M-CTRL	M-HFS
Plasma fasting glycemia (mM)	4.72 ± 0.2	5.77 ± 0.02 *	5.16 ± 0.2	5.88 ± 0.2 †
Plasma HDL-Cholesterol (mM)	0.5 ± 0.02	0.45 ± 0.04	0.39 ± 0.02	0.46 ± 0.03
Plasma LDL-Cholesterol (mM)	0.29 ± 0.03	0.48 ± 0.04 *^,^‡	0.26 ± 0.01	0.31 ± 0.03
Plasma Free fatty acids (mM)	0.11 ± 0.02	0.19 ± 0.04	0.15 ± 0.02	0.18 ± 0.02
Fat mass (%)	9.92 ± 0.9	18.03 ± 2.1 *	10.4 ± 0.5	21.4 ± 1.5 †
Visceral adipose tissue (g)	16.54 ± 1.82	41.93 ± 6.98	30.28 ±1.74	97.36 ± 17.64 †
Subcutaneous adipose tissue (g)	8.66 ± 1.03	20.76 ± 4.78	21.51 ± 2.72	51.19 ± 9.09 †
Heart weight (g)	0.75 ± 0.02	0.80 ± 0.02	1.22 ± 0.02	1.45 ± 0.03 †
Tibia length (cm)	3.49 ± 0.05	3.48 ± 0.04	4.04 ± 0.03	4.08 ± 0.03
Heart/Tibia (g/cm)	0.21 ± 0.004	0.23 ± 0.006	0.30 ± 0.01	0.36 ± 0.01 †
Systolic blood pressure (mmHg)	112 ± 3.3	128 ± 10.4	134 ± 4.3	143 ± 3.9
Diastolic blood pressure (mmHg)	76 ± 2.9	90 ± 8.1	97 ± 3.1	101 ± 3.2
Plasma 8-iso-PGF2α (pg/mL)	4673 ± 567	4692 ± 458	4109 ± 556	4487 ± 218

One-way ANOVA was performed for all parameters. A p-value of less than or equal to 0.05 was considered to indicate significant difference. Data are means ± SEM. * *p* < 0.05 F-HFS vs. F-CTRL; † *p* < 0.05 M-HFS vs. M-CTRL; ‡ *p* < 0.05 F-HFS vs. M-HFS. F-CTRL: female control; F-HFS: female high-fat high-sucrose; M-CTRL: male control; M-HFS: male high-fat high-sucrose.

## Data Availability

The data that support the findings of this study are available from the corresponding author, [N.F.], upon reasonable request.

## Abbreviations

^31^P-MRS: phosphorus-31 magnetic resonance spectroscopy; CTRL: control; CV: cardiovascular; DP: developed pressure; F-CTRL: female control; HFS: high-fat high-sucrose; F-HFS female high-fat high-sucrose; HR: heart rate; IPGTT: intraperitoneal glucose tolerance test; M-CTRL: male control; MDA: malondialdehyde; M-HFS: male high-fat high-sucrose; pHi: intracellular pH.
